# A study of awareness on HIV/AIDS among adolescents: A Longitudinal Study on UDAYA data

**DOI:** 10.1038/s41598-021-02090-9

**Published:** 2021-11-24

**Authors:** Shobhit Srivastava, Shekhar Chauhan, Ratna Patel, Pradeep Kumar

**Affiliations:** 1 Ph.D. Research Scholar, Department of Survey Research & Data Analytics, International Institute for Population Sciences, Mumbai, India; 2 Ph.D. Research Scholar, Department of Family and Generations, International Institute for Population Sciences, Mumbai, India; 3 Ph.D. Research Scholar, Department of Public Health and Mortality Studies, International Institute for Population Sciences, Mumbai, India

**Keywords:** Health care, Health services, Public health

## Abstract

Acquired Immunodeficiency Syndrome caused by Human Immunodeficiency Virus (HIV) poses a severe challenge to healthcare and is a significant public health issue worldwide. This study intends to examine the change in the awareness level of HIV among adolescents. Furthermore, this study examined the factors associated with the change in awareness level on HIV-related information among adolescents over the period. Data used for this study were drawn from Understanding the lives of adolescents and young adults, a longitudinal survey on adolescents aged 10–19 in Bihar and Uttar Pradesh. The present study utilized a sample of 4421 and 7587 unmarried adolescent boys and girls, respectively aged 10–19 years in wave-1 and wave-2. Descriptive analysis and t-test and proportion test were done to observe changes in certain selected variables from wave-1 (2015–2016) to wave-2 (2018–2019). Moreover, random effect regression analysis was used to estimate the association of change in HIV awareness among unmarried adolescents with household and individual factors. The percentage of adolescent boys who had awareness regarding HIV increased from 38.6% in wave-1 to 59.9% in wave-2. Among adolescent girls, the percentage increased from 30.2 to 39.1% between wave-1 & wave-2. With the increase in age and years of schooling, the HIV awareness increased among adolescent boys ([Coef: 0.05; *p* < 0.01] and [Coef: 0.04; *p* < 0.01]) and girls ([Coef: 0.03; *p* < 0.01] and [Coef: 0.04; *p* < 0.01]), respectively. The adolescent boys [Coef: 0.06; *p* < 0.05] and girls [Coef: 0.03; *p* < 0.05] who had any mass media exposure were more likely to have an awareness of HIV. Adolescent boys' paid work status was inversely associated with HIV awareness [Coef: − 0.01; *p* < 0.10]. Use of internet among adolescent boys [Coef: 0.18; *p* < 0.01] and girls [Coef: 0.14; *p* < 0.01] was positively associated with HIV awareness with reference to their counterparts. There is a need to intensify efforts in ensuring that information regarding HIV should reach vulnerable sub-groups, as outlined in this study. It is important to mobilize the available resources to target the less educated and poor adolescents, focusing on rural adolescents.

## Introduction

Acquired Immunodeficiency Syndrome (AIDS) caused by Human Immunodeficiency Virus (HIV) poses a severe challenge to healthcare and is a significant public health issue worldwide. So far, HIV has claimed almost 33 million lives; however, off lately, increasing access to HIV prevention, diagnosis, treatment, and care has enabled people living with HIV to lead a long and healthy life^1^. By the end of 2019, an estimated 38 million people were living with HIV^1^. More so, new infections fell by 39 percent, and HIV-related deaths fell by almost 51 percent between 2000 and 2019^1^. Despite all the positive news related to HIV, the success story is not the same everywhere; HIV varies between region, country, and population, where not everyone is able to access HIV testing and treatment and care^1^. HIV/AIDS holds back economic growth by destroying human capital by predominantly affecting adolescents and young adults^2^.

There are nearly 1.2 billion adolescents (10–19 years) worldwide, which constitute 18 percent of the world’s population, and in some countries, adolescents make up as much as one-fourth of the population^3^. In India, adolescents comprise more than one-fifth (21.8%) of the total population^4^. Despite a decline projection for the adolescent population in India^5^, there is a critical need to hold adolescents as adolescence is characterized as a period when peer victimization/pressure on psychosocial development is noteworthy^6^. Peer victimization/pressure is further linked to risky sexual behaviours among adolescents^7,8^. A higher proportion of low literacy in the Indian population leads to a low level of awareness of HIV/AIDS^9^. Furthermore, the awareness of HIV among adolescents is quite alarming^10–12^.

Unfortunately, there is a shortage of evidence on what predicts awareness of HIV among adolescents. Almost all the research in India is based on beliefs, attitudes, and awareness of HIV among adolescents^2,12^. However, few other studies worldwide have examined mass media as a strong predictor of HIV awareness among adolescents^13^. Mass media is an effective channel to increase an individuals’ knowledge about sexual health and improve understanding of facilities related to HIV prevention^14,15^. Various studies have outlined other factors associated with the increasing awareness of HIV among adolescents, including; age^16–18^, occupation^18^, education^16–19^, sex^16^, place of residence^16^, marital status^16^, and household wealth index^16^.

Several community-based studies have examined awareness of HIV among Indian adolescents^2,10,12,20–22^. However, studies investigating awareness of HIV among adolescents in a larger sample size remained elusive to date, courtesy of the unavailability of relevant data. Furthermore, no study in India had ever examined awareness of HIV among adolescents utilizing information on longitudinal data. To the author’s best knowledge, this is the first study in the Indian context with a large sample size that examines awareness of HIV among adolescents and combines information from a longitudinal survey. Therefore, this study intends to examine the change in the awareness level of HIV among adolescents. Furthermore, this study examined the factors associated with a change in awareness level on HIV-related information among adolescents over the period.

## Data and methods

### Data

Data used for this study were drawn from Understanding the lives of adolescents and young adults (UDAYA), a longitudinal survey on adolescents aged 10–19 in Bihar and Uttar Pradesh^23^. The first wave was conducted in 2015–2016, and the follow-up survey was conducted after three years in 2018–2019^23^. The survey provides the estimates for state and the sample of unmarried boys and girls aged 10–19 and married girls aged 15–19. The study adopted a systematic, multi-stage stratified sampling design to draw sample areas independently for rural and urban areas. 150 primary sampling units (PSUs)—villages in rural areas and census wards in urban areas—were selected in each state, using the 2011 census list of villages and wards as the sampling frame. In each primary sampling unit (PSU), households to be interviewed were selected by systematic sampling. More details about the study design and sampling procedure have been published elsewhere^23^. Written consent was obtained from the respondents in both waves. In wave 1 (2015–2016), 20,594 adolescents were interviewed using the structured questionnaire with a response rate of 92%.

Moreover, in wave 2 (2018–2019), the study interviewed the participants who were successfully interviewed in 2015–2016 and who consented to be re-interviewed^23^. Of the 20,594 eligible for the re-interview, the survey re-interviewed 4567 boys and 12,251 girls (married and unmarried). After excluding the respondents who gave an inconsistent response to age and education at the follow-up survey (3%), the final follow-up sample covered 4428 boys and 11,864 girls with the follow-up rate of 74% for boys and 81% for girls. The effective sample size for the present study was 4421 unmarried adolescent boys aged 10–19 years in wave-1 and wave-2. Additionally, 7587 unmarried adolescent girls aged 10–19 years were interviewed in wave-1 and wave-2^23^. The cases whose follow-up was lost were excluded from the sample to strongly balance the dataset and set it for longitudinal analysis using xtset command in STATA 15. The survey questionnaire is available at https://dataverse.harvard.edu/file.xhtml?fileId=4163718&version=2.0 & https://dataverse.harvard.edu/file.xhtml?fileId=4163720&version=2.0.

#### Outcome variable

HIV awareness was the outcome variable for this study, which is dichotomous. The question was asked to the adolescents ‘Have you heard of HIV/AIDS?’ The response was recorded as yes and no.

#### Exposure variables

The predictors for this study were selected based on previous literature. These were age (10–19 years at wave 1, continuous variable), schooling (continuous), any mass media exposure (no and yes), paid work in the last 12 months (no and yes), internet use (no and yes), wealth index (poorest, poorer, middle, richer, and richest), religion (Hindu and Non-Hindu), caste (Scheduled Caste/Scheduled Tribe, Other Backward Class, and others), place of residence (urban and rural), and states (Uttar Pradesh and Bihar).

Exposure to mass media (how often they read newspapers, listened to the radio, and watched television; responses on the frequencies were: almost every day, at least once a week, at least once a month, rarely or not at all; adolescents were considered to have any exposure to mass media if they had exposure to any of these sources and as having no exposure if they responded with ‘not at all’ for all three sources of media)^24^. Household wealth index based on ownership of selected durable goods and amenities with possible scores ranging from 0 to 57; households were then divided into quintiles, with the first quintile representing households of the poorest wealth status and the fifth quintile representing households with the wealthiest status^25^.

#### Statistical analysis

Descriptive analysis was done to observe the characteristics of unmarried adolescent boys and girls at wave-1 (2015–2016). In addition, the changes in certain selected variables were observed from wave-1 (2015–2016) to wave-2 (2018–2019), and the significance was tested using t-test and proportion test^26,27^. Moreover, random effect regression analysis^28,29^ was used to estimate the association of change in HIV awareness among unmarried adolescents with household factors and individual factors. The random effect model has a specific benefit for the present paper's analysis: its ability to estimate the effect of any variable that does not vary within clusters, which holds for household variables, e.g., wealth status, which is assumed to be constant for wave-1 and wave-2^30^.

## Results

Table 1 represents the socio-economic profile of adolescent boys and girls. The estimates are from the baseline dataset, and it was assumed that none of the household characteristics changed over time among adolescent boys and girls.

**Table 1:** Socio-economic characteristics of study population, 2015–2016.

Background characteristics	Adolescent boys		Adolescent girls	
	Sample	Percentage	Sample	Percentage
**Wealth Index**				
Poorest	414	11.4	756	12.3
Poorer	699	19.9	1065	17.2
Middle	908	22.3	1476	21.1
Richer	1195	23.8	2118	24.9
Richest	1212	22.7	2192	24.4
**Religion**				
Hindu	3729	84.9	5674	76.7
Non-Hindu	699	15.1	1933	23.3
**Caste**				
SC/ST	1080	27.5	1561	23.3
OBC	2518	54.8	4403	55.1
Others	830	17.8	1643	21.6
**Place of residence**				
Urban	1989	16.8	3424	17.0
Rural	2439	83.2	4183	83.0
**States**				
Uttar Pradesh	2300	63.9	4135	72.2
Bihar	2128	32.1	3472	27.8
Total	4428	100.0	7607	100.0

*SC/ST:* Scheduled Caste/Scheduled Tribe; *OBC:* Other Backward Class.

**Figure 1:**
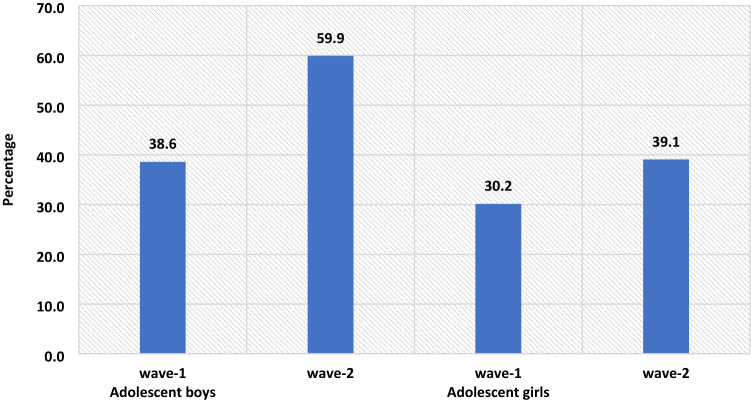
The percenate of HIV awareness among adolescent boys and girls, wave-1 (2015–2016) and wave-2 (2018–2019).

Figure 1 represents the change in HIV awareness among adolescent boys and girls. The percentage of adolescent boys who had awareness regarding HIV increased from 38.6% in wave-1 to 59.9% in wave-2. Among adolescent girls, the percentage increased from 30.2% in wave-1 to 39.1% in wave-2.

Table 2 represents the summary statistics for explanatory variables used in the analysis of UDAYA wave-1 and wave-2. The exposure to mass media is almost universal for adolescent boys, while for adolescent girls, it increases to 93% in wave-2 from 89.8% in wave-1. About 35.3% of adolescent boys were engaged in paid work during wave-1, whereas in wave-II, the share dropped to 33.5%, while in the case of adolescent girls, the estimates are almost unchanged. In wave-1, about 27.8% of adolescent boys were using the internet, while in wave-2, there is a steep increase of nearly 46.2%. Similarly, in adolescent girls, the use of the internet increased from 7.6% in wave-1 to 39.3% in wave-2.

**Table 2:** Summary statistics for explanatory variables used in the analysis of UDAYA wave-1 and wave-2.

Variables	Adolescent boys		*p* value	Adolescent girls		*p* value
	Wave-1	Wave-2		Wave-1	Wave-2	
Mean age (years)	14.8	17.8	<0.001	15.8	18.8	<0.001
Mean schooling (years)	7.4	9.3	<0.001	8.0	9.5	<0.001
Any mass media	97.5	98.0	0.028	89.8	93.0	<0.001
Paid work in last 12 months	35.3	33.5	0.648	22.5	22.9	0.613
Internet use	27.8	74.0	<0.001	7.6	39.3	<0.001
Sample (N)	4428	4428		7607	7607	

Table 3 represents the estimates from random effects for awareness of HIV among adolescent boys and girls. It was found that with the increases in age and years of schooling the HIV awareness increased among adolescent boys ([Coef: 0.05; *p* < 0.01] and [Coef: 0.04; *p* < 0.01]) and girls ([Coef: 0.03; *p* < 0.01] and [Coef: 0.04; *p* < 0.01]), respectively. The adolescent boys [Coef: 0.06; *p* < 0.05] and girls [Coef: 0.03; *p* < 0.05] who had any mass media exposure were more likely to have an awareness of HIV in comparison to those who had no exposure to mass media. Adolescent boys' paid work status was inversely associated with HIV awareness about adolescent boys who did not do paid work [Coef: − 0.01; *p* < 0.10]. Use of the internet among adolescent boys [Coef: 0.18; *p* < 0.01] and girls [Coef: 0.14; *p* < 0.01] was positively associated with HIV awareness in reference to their counterparts.

**Table 3:** Estimated effects of explanatory variables on the awareness of HIV from random effect models.

Variables	Adolescent boys (10–19) at wave-1	Adolescent girls (10–19) at wave-1
	Random effect Coefficient (CI)	Random effect Coefficient (CI)
**Individual characteristics**		
Age (years)	0.05*** (0.05, 0.06)	0.03*** (0.02, 0.03)
Schooling (years)	0.04*** (0.04, 0.05)	0.04*** (0.04, 0.05)
**Any mass media exposure**		
No	Ref	Ref
Yes	0.06** (0.01, 0.13)	0.03** (0.01, 0.06)
**Paid work**		
No	Ref	Ref
Yes	− 0.01* (− 0.03, 0.01)	0.01 (− 0.01, 0.02)
**Internet use**		
No	Ref	Ref
Yes	0.18*** (0.16, 0.20)	0.14*** (0.12, 0.16)
**Household characteristics**		
**Wealth Index**		
Poorest	Ref	Ref
Poorer	0.04** (0.00, 0.07)	0.00 (− 0.03, 0.03)
Middle	0.05*** (0.02, 0.09)	0.03** (0.00, 0.06)
Richer	0.08*** (0.04, 0.11)	0.10*** (0.07, 0.13)
Richest	0.12*** (0.09, 0.16)	0.19*** (0.16, 0.22)
**Religion**		
Hindu	Ref	Ref
Non-Hindu	− 0.01 (− 0.03, 0.02)	− 0.09*** (− 0.11, − 0.08)
**Caste**		
Others	Ref	Ref
SC/ST	− 0.01 (− 0.04, 0.02)	0.00 (− 0.02, 0.02)
OBC	− 0.01 (− 0.03, 0.02)	0.04*** (0.02, 0.07)
**Place of residence**		
Urban	Ref	Ref
Rural	− 0.03*** (− 0.05, − 0.02)	− 0.09*** (− 0.11, − 0.08)
**States**		
Uttar Pradesh	Ref	Ref
Bihar	0.04*** (0.03, 0.06)	0.02*** (0.01, 0.04)
**Year**		
2015–2016	Ref	Ref
2018–2019	− 0.10*** (− 0.12, − 0.08)	− 0.10*** (− 0.12, − 0.09)
sigma_u	0.145	0.231
sigma_e	0.345	0.325
rho	0.150	0.335

**if *p* < 0.05 ***if *p* < 0.001; *Ref* Reference; *CI* confidence interval; *SC/ST* Scheduled Caste/Scheduled Tribe; OBC: Other Backward Class.

The awareness regarding HIV increases with the increase in household wealth index among both adolescent boys and girls. The adolescent girls from the non-Hindu household had a lower likelihood to be aware of HIV in reference to adolescent girls from Hindu households [Coef: − 0.09; *p* < 0.01]. Adolescent girls from non-SC/ST households had a higher likelihood of being aware of HIV in reference to adolescent girls from other caste households [Coef: 0.04; *p* < 0.01]. Adolescent boys [Coef: − 0.03; *p* < 0.01] and girls [Coef: − 0.09; *p* < 0.01] from a rural place of residence had a lower likelihood to be aware about HIV in reference to those from the urban place of residence. Adolescent boys [Coef: 0.04; *p* < 0.01] and girls [Coef: 0.02; *p* < 0.01] from Bihar had a higher likelihood to be aware about HIV in reference to those from Uttar Pradesh.

## Discussion

This is the first study of its kind to address awareness of HIV among adolescents utilizing longitudinal data in two indian states. Our study demonstrated that the awareness of HIV has increased over the period; however, it was more prominent among adolescent boys than in adolescent girls. Overall, the knowledge on HIV was relatively low, even during wave-II. Almost three-fifths (59.9%) of the boys and two-fifths (39.1%) of the girls were aware of HIV. The prevalence of awareness on HIV among adolescents in this study was lower than almost all of the community-based studies conducted in India^10,11,22^. A study conducted in slums in Delhi has found almost similar prevalence (40% compared to 39.1% during wave-II in this study) of awareness of HIV among adolescent girls^31^. The difference in prevalence could be attributed to the difference in methodology, study population, and study area.

The study found that the awareness of HIV among adolescent boys has increased from 38.6 percent in wave-I to 59.9 percent in wave-II; similarly, only 30.2 percent of the girls had an awareness of HIV during wave-I, which had increased to 39.1 percent. Several previous studies corroborated the finding and noticed a higher prevalence of awareness on HIV among adolescent boys than in adolescent girls^16,32–34^. However, a study conducted in a different setting noticed a higher awareness among girls than in boys^35^. Also, a study in the Indian context failed to notice any statistical differences in HIV knowledge between boys and girls^18^. Gender seems to be one of the significant determinants of comprehensive knowledge of HIV among adolescents. There is a wide gap in educational attainment among male and female adolescents, which could be attributed to lower awareness of HIV among girls in this study. Higher peer victimization among adolescent boys could be another reason for higher awareness of HIV among them^36^. Also, cultural double standards placed on males and females that encourage males to discuss HIV/AIDS and related sexual matters more openly and discourage or even restrict females from discussing sexual-related issues could be another pertinent factor of higher awareness among male adolescents^33^. Behavioural interventions among girls could be an effective way to improving knowledge HIV related information, as seen in previous study^37^. Furthermore, strengthening school-community accountability for girls' education would augment school retention among girls and deliver HIV awareness to girls^38^.

Similar to other studies^2,10,17,18,39–41^, age was another significant determinant observed in this study. Increasing age could be attributed to higher education which could explain better awareness with increasing age. As in other studies^18,39,41–46^, education was noted as a significant driver of awareness of HIV among adolescents in this study. Higher education might be associated with increased probability of mass media and internet exposure leading to higher awareness of HIV among adolescents. A study noted that school is one of the important factors in raising the awareness of HIV among adolescents, which could be linked to higher awareness among those with higher education^47,48^. Also, schooling provides adolescents an opportunity to improve their social capital, leading to increased awareness of HIV.

Following previous studies^18,40,46^, the current study also outlines a higher awareness among urban adolescents than their rural counterparts. One plausible reason for lower awareness among adolescents in rural areas could be limited access to HIV prevention information^16^. Moreover, rural–urban differences in awareness of HIV could also be due to differences in schooling, exposure to mass media, and wealth^44,45^. The household's wealth status was also noted as a significant predictor of awareness of HIV among adolescents. Corroborating with previous findings^16,33,42,49^, this study reported a higher awareness among adolescents from richer households than their counterparts from poor households. This could be because wealthier families can afford mass-media items like televisions and radios for their children, which, in turn, improves awareness of HIV among adolescents^33^.

Exposure to mass media and internet access were also significant predictors of higher awareness of HIV among adolescents. This finding agrees with several previous research, and almost all the research found a positive relationship between mass-media exposure and awareness of HIV among adolescents^10^. Mass media addresses such topics more openly and in a way that could attract adolescents’ attention is the plausible reason for higher awareness of HIV among those having access to mass media and the internet^33^. Improving mass media and internet usage, specifically among rural and uneducated masses, would bring required changes. Integrating sexual education into school curricula would be an important means of imparting awareness on HIV among adolescents; however, this is debatable as to which standard to include the required sexual education in the Indian schooling system. Glick (2009) thinks that the syllabus on sexual education might be included during secondary schooling^44^. Another study in the Indian context confirms the need for sex education for adolescents^50,51^.

## Limitations and strengths of the study

The study has several limitations. At first, the awareness of HIV was measured with one question only. Given that no study has examined awareness of HIV among adolescents using longitudinal data, this limitation is not a concern. Second, the study findings cannot be generalized to the whole Indian population as the study was conducted in only two states of India. However, the two states selected in this study (Uttar Pradesh and Bihar) constitute almost one-fourth of India’s total population. Thirdly, the estimates were provided separately for boys and girls and could not be presented combined. However, the data is designed to provide estimates separately for girls and boys. The data had information on unmarried boys and girls and married girls; however, data did not collect information on married boys. Fourthly, the study estimates might have been affected by the recall bias. Since HIV is a sensitive topic, the possibility of respondents modifying their responses could not be ruled out. Hawthorne effect, respondents, modifying aspect of their behaviour in response, has a role to play in HIV related study^52^. Despite several limitations, the study has specific strengths too. This is the first study examining awareness of HIV among adolescent boys and girls utilizing longitudinal data. The study was conducted with a large sample size as several previous studies were conducted in a community setting with a minimal sample size^10,12,18,20,53^.

## Conclusion

The study noted a higher awareness among adolescent boys than in adolescent girls. Specific predictors of high awareness were also noted in the study, including; higher age, higher education, exposure to mass media, internet use, household wealth, and urban residence. Based on the study findings, this study has specific suggestions to improve awareness of HIV among adolescents. There is a need to intensify efforts in ensuring that information regarding HIV should reach vulnerable sub-groups as outlined in this study. It is important to mobilize the available resources to target the less educated and poor adolescents, focusing on rural adolescents. Investment in education will help, but it would be a long-term solution; therefore, public information campaigns could be more useful in the short term.
